# Observation of mechanical bound states in the continuum in an optomechanical microresonator

**DOI:** 10.1038/s41377-022-00971-w

**Published:** 2022-11-18

**Authors:** Yue Yu, Xiang Xi, Xiankai Sun

**Affiliations:** Department of Electronic Engineering, The Chinese University of Hong Kong, Shatin, New Territories, Hong Kong SAR, China

**Keywords:** Integrated optics, Photonic devices, Microresonators

## Abstract

Bound states in the continuum (BICs) are a type of waves that are perfectly confined in the continuous spectrum of radiating waves without interaction with them. Here, we fabricated, with CMOS-compatible processes on a silicon chip, a wheel-shaped optomechanical microresonator, in which we experimentally observed the BIC in the micromechanical domain. The BIC results from destructive interference between two dissipative mechanical modes of the microresonator under broken azimuthal symmetry. Such BICs can be obtained from devices with large and robust supporting structures with variable sizes, which substantially reduces fabrication difficulty and allows for versatile application environments. Our results open a new way of phonon trapping in micromechanical structures with dissipation channels, and produce long phonon lifetimes that are desired in many mechanical applications such as mechanical oscillators, sensors, and quantum information processors.

## Abstract (web summary)

Mechanical bound states in the continuum are observed in an optomechanical microresonator, which open a new way of phonon trapping in micromechanical structures with dissipation channels.

## Graphical abstract

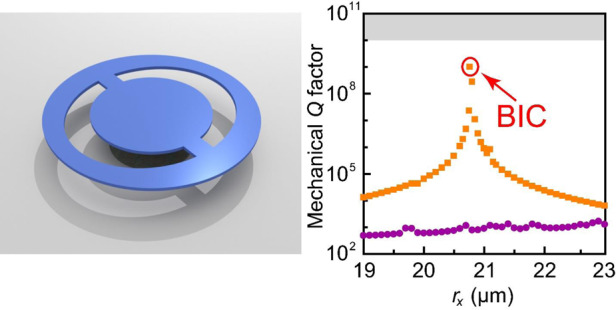

## Introduction

Micro- and nanomechanical resonators, which possess a very small mass and can be strongly coupled to light and matter, have been explored for precision metrology applications like mass and force sensing^1^ and employed for investigating macroscopic quantum physics^2,3^. Reducing mechanical dissipation is crucial to these applications since it allows enhanced mechanical fields with long coherence time and thus leads to improved performance. The conventional wisdom of reducing the dissipation loss relies on separating their eigenmodes from the continuum of lossy modes by constructing deep energy potentials with different materials or periodic structures^4,5^. For another type of nonperiodic individual resonators, where the bandgap shielding strategy cannot be applied, reducing the dissipation loss relies on minimizing their supporting structures^6,7^, which increases device fabrication difficulty and sets restrictions on their application areas. For example, devices based on such delicate mechanical structures cannot be used repeatedly for fluid-based applications, because they would likely fail when the ambient environment changes from a liquid to a gas.

Bound states in the continuum (BICs) refer to a type of eigenstates with infinite lifetime yet spectrally overlapping with lossy states in the continuum^8^. Originally introduced to quantum mechanics, the concept of BICs has been extended to optical^9–14^, acoustic^15–18^, and mechanical^19,20^ domains, and enabled many unprecedented applications such as low-threshold lasing^21–23^, ultrasensitive sensing^24^, and vortex beam generation^25^. To date, most experimental demonstrations of BICs in optics and mechanics are based on periodic structures with certain symmetry^26–30^. These devices usually have a large footprint with a large modal volume or effective mass, which sets limitations to their application scenarios. In contrast to devices based on periodic structures, nonperiodic individual optical and mechanical resonators can more easily have confined fields with strong modal intensity at the micro/nanoscale, leading to a series of applications in precision metrology as well as studies of macroscopic quantum physics. BICs in individual optical^31,32^ and acoustic^33–36^ resonators have been demonstrated. However, experimental demonstration of BICs in an individual mechanical resonator remains elusive.

Here, we experimentally demonstrated mechanical BICs in an optomechanical microresonator. By breaking the azimuthal symmetry, we introduced coupling between a radial-contour mode and a wine-glass mode of a wheel-shaped structure to obtain destructive interference between energy dissipation of the two modes, which produces a mechanical BIC under the Friedrich–Wintgen condition^37^. The mechanical BIC was experimentally confirmed by optomechanical measurement of the devices in vacuum. In contrast to conventional BICs requiring certain symmetry, the demonstrated mechanical BICs represent a new paradigm for constructing high-*Q* micromechanical resonators through symmetry breaking^38^. In addition, the low-loss mechanical BIC has high tolerance on the supporting rods’ width from hundreds of nanometers to several micrometers.

## Results

**Fig. 1:**
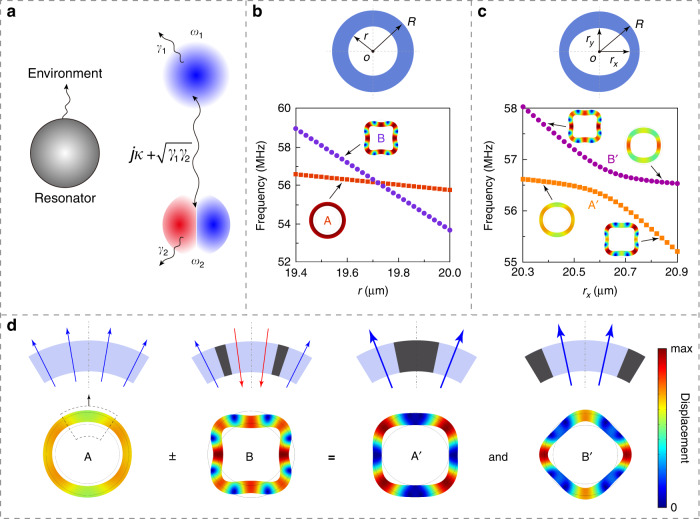
Construction of mechanical BIC in a micromechanical resonator based on modal coupling. **a** Schematic of dispersive and dissipative coupling between two eigenmodes of a resonating system. **b** Simulated frequencies of mode A (dark red rectangles) and mode B (violet dots) of a ring-shaped silicon micromechanical resonator with azimuthal symmetry as a function of the inner radius *r*. The resonator has a thickness *h* = 220 nm and outer radius *R* = 26.1 μm. Modes A and B are the fundamental radial-contour mode and the 4th-order wine-glass mode, respectively. **c** Simulated frequencies of mode A’ (orange rectangles) and mode B' (purple dots) of a ring-shaped resonator with broken azimuthal symmetry as a function of semi-major axis of the inner boundary *r*_*x*_. The resonator has a thickness *h* = 220 nm, outer radius *R* = 26.1 μm, and semi-minor axis of the inner boundary *r*_*y*_ = 18.7 μm. **d** Generation of the hybrid modes A’ and B’ from coupling of the original modes A and B at the anticrossing point

To construct BICs in an individual mechanical resonator, suppose we have a resonator supporting two dissipative modes coupled with each other, as shown in Fig. 1a. Such a system can be described by a Hamiltonian

1 $$H = \left( {\begin{array}{*{20}{c}} {\omega _1 - j\gamma _1} & {\kappa - j\sqrt {\gamma _1\gamma _2} } \\ {\kappa - j\sqrt {\gamma _1\gamma _2} } & {\omega _2 - j\gamma _2} \end{array}} \right)$$

where *ω*_1_ (*γ*_1_) and *ω*_2_ (*γ*_2_) are the resonant frequencies (dissipation rates) of the two modes. The two modes are coupled with each other with a coupling coefficient *κ*, which results in an anticrossing of these two modes. At this anticrossing point, when the Friedrich–Wintgen condition

2 $$\kappa \left( {\gamma _1 - \gamma _2} \right){{{\mathrm{ = }}}}\sqrt {\gamma _1\gamma _2} \left( {\omega _1 - \omega _2} \right)$$

is satisfied^13,39^, the complex resonant frequencies become (see Supplementary Information, Section S1)

3 $$\Omega _1 = \frac{{\omega _1 + \omega _2}}{2} + \frac{{\kappa \left( {\gamma _1 + \gamma _2} \right)}}{{2\sqrt {\gamma _1\gamma _2} }} - j\left( {\gamma _1 + \gamma _2} \right)$$

4 $$\Omega _2 = \frac{{\omega _1 + \omega _2}}{2} - \frac{{\kappa \left( {\gamma _1 + \gamma _2} \right)}}{{2\sqrt {\gamma _1\gamma _2} }}$$

As shown in Eq. (4), Ω_2_ has a vanishing imaginary part, which means that this hybrid mode experiences zero dissipation loss and thus can be a BIC. In this system, when one of the two hybrid modes becomes lossless, the Friedrich–Wintgen condition is also satisfied (See Supplementary Information, Section S2). Therefore, one can verify a Friedrich–Wintgen BIC by measuring the dissipation loss of the two hybrid modes of the system.

First, we consider a ring-shaped thin-plate micromechanical resonator as shown in Fig. 1b. It is made in 220-nm-thick silicon and has an inner radius *r* and an outer radius *R* (*r*, *R* ≫ 220 nm). Such resonators support two types of in-plane mechanical modes: radial-contour modes and wine-glass modes. Since these two types of modes have different dependence on *r*, fixing *R* = 26.1 µm and varying *r* lead to a crossing of resonant frequencies of the fundamental radial-contour mode (mode A in Fig. 1b) and the 4th-order wine-glass mode (mode B in Fig. 1b). In a ring-shaped resonator with perfect azimuthal symmetry, mode A and mode B are orthogonal to each other without modal coupling. Therefore, the Friedrich–Wintgen condition in Eq. (2) for BICs cannot be satisfied. To introduce modal coupling for satisfying the Friedrich–Wintgen condition, we break the azimuthal symmetry of the ring-shaped resonator by modifying its inner boundary to an ellipse, with semi-major and semi-minor axes being respectively *r*_*x*_ and *r*_*y*_, as shown in Fig. 1c. Figure 1c also plots the simulated modal frequencies of the modified structure as a function of *r*_*x*_ with fixed *r*_*y*_ = 18.7 μm and *R* = 26.1 μm, where an anticrossing occurs near *r*_*x*_ = 20.6 μm indicating the coupling between the two mechanical modes. At the anticrossing point, the energy exchange between the original mode A and mode B leads to two hybrid modes, namely mode A' and mode B', as shown in Fig. 1d. Note that although the structure in Fig. 1c can have the required coupling between different modes which can support a BIC, a realistic device must also include supporting structures that are connected to the substrate, which act as the dissipation channel for both mechanical modes. Compared with the original modes A and B, the hybrid modes A' and B' have larger regions where the modal displacement is near zero (Fig. 1d). Therefore, by attaching the supporting structures to these regions, it is possible to reduce energy dissipation of the ring-shaped mechanical resonator to the substrate.

**Fig. 2:**
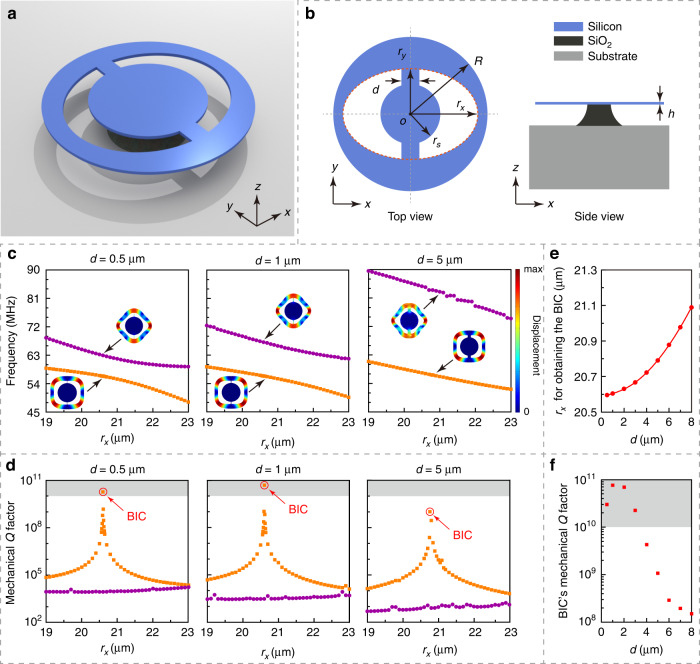
Design and numerical simulation of mechanical BIC in a micromechanical resonator. **a** Illustration of the wheel-shaped micromechanical resonator that supports mechanical BIC. **b** Top and side views of the micromechanical resonator, with dimension labels. **c** Simulated frequencies of modes A' and B' of the micromechanical resonator as a function of *r*_*x*_ for *d* = 0.5, 1, and 5 μm. The insets show the corresponding modal displacement profiles. **d** Simulated mechanical *Q* factors of modes A' and B' as a function of *r*_*x*_ for *d* = 0.5, 1, and 5 μm. The BIC point where the highest mechanical *Q* factor is achieved is marked in each plot. **e** The value of *r*_*x*_ for obtaining the BIC as a function of *d*. **f** Simulated mechanical *Q* factor of the BIC as a function of *d*. In (**c**–**f**), the fixed geometric parameters of the micromechanical resonator are *r*_*y*_ = 18.7 μm, *R* = 26.1 μm, *r*_*s*_ = 14.7 μm, and *h* = 220 nm. In (**d)** and (**f**), the gray areas indicate the regimes reaching the limit of numerical simulation, where the simulated results do not converge

Next, we investigate a realistic structure in which two supporting rods are added to the azimuthal-symmetry-broken ring-shaped resonator making a wheel-shaped resonator as shown in Fig. 2a, b. We need to engineer this structure and analyze the modal coupling to satisfy the Friedrich–Wintgen condition [Eq. (2)] for constructing a mechanical BIC. Figure 2a is a three-dimensional view of the entire device structure where the wheel-shaped resonator is seated on a silicon oxide (SiO_2_) pedestal on the substrate. Figure 2b shows the top and side views of the entire device, where the wheel-shaped silicon micromechanical resonator, the SiO_2_ pedestal, and the substrate are marked in blue, black, and gray, respectively. The additional two parameters for the wheel-shaped resonator *d* and *r*_*s*_ are the supporting rods’ width and the center disk radius, respectively. To investigate the influence of the supporting rods on the modal coupling, we simulated the frequencies and mechanical *Q* factors of mode A' and mode B' as a function of the semi-major axis *r*_*x*_ for different rod widths *d*, with the results shown in Fig. 2c, d. The other geometric parameters are fixed at *r*_*y*_ = 18.7 μm, *R* = 26.1 μm, and *r*_*s*_ = 14.7 μm. The insets in Fig. 2c show the displacement profiles of the corresponding mechanical modes. It can be found that an anticrossing in the modal frequencies (Fig. 2c) and a drastic variation in the mechanical *Q* factor of mode A' (Fig. 2d) occur simultaneously near *r*_*x*_ = 20.8 μm, despite a large variation of *d* from 0.5 to 5 μm. These behaviors indicate that mode A' becomes a Friedrich–Wintgen quasi-BIC^37^. Note that the high-*Q* Friedrich–Wintgen BIC can be obtained from the wheel-shaped resonator with *d* as large as several micrometers. One reason is that the hybrid mode A' has a larger region of near-zero displacement than the original uncoupled wine-glass mode (mode B). Actually, we simulated a series of structures with *d* varying from 0.5 to 8 μm and collected the *r*_*x*_ value and mechanical *Q* factor when the BIC is achieved (marked by the red circles in Fig. 2d), with the results plotted in Fig. 2e, f, respectively. Figure 2f shows that the simulated mechanical *Q* factor of the BIC can maintain above 10^8^ in such a wide range of *d* from 0.5 to 8 μm (See Supplementary Information, Section S5), demonstrating excellent robustness against variations of the width of the dissipation channel. Compared with conventional mechanical systems which rely on minimized supporting rods^6,7^ or surrounding phononic bandgap structures^5^ for reducing the clamping loss and achieving high mechanical *Q* factors, the Friedrich–Wintgen BIC can exist in mechanical resonators with simply designed sturdy supporting structures, which substantially alleviate device fabrication difficulty, facilitate thermalization and heat dissipation, and enable device applications in versatile environments.

**Fig. 3:**
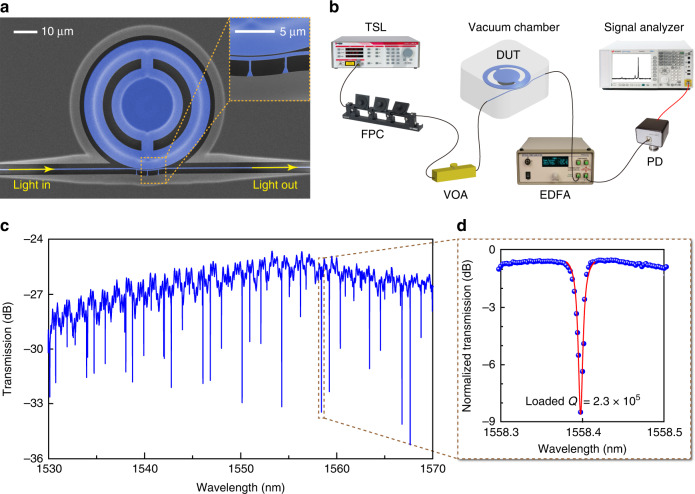
Device fabrication and experimental characterization. **a** Scanning electron microscope image of a fabricated silicon optomechanical microresonator. The nearby bus waveguide is used for coupling light into the resonator for optical measurement of its mechanical modes. The inset is a close-up showing the details in the coupling region of the resonator and bus waveguide. **b** Experimental setup. TSL, tunable semiconductor laser; FPC, fiber polarization controller; VOA, variable optical attenuator; DUT, device under test; EDFA, erbium-doped fiber amplifier; PD, photodetector. **c** Measured optical transmission spectrum of the device in (**a**). **d** Zoomed-in optical transmission spectrum showing an optical resonance with Lorentzian-fitted optical *Q* factor

To measure the mechanical BIC, we fabricated the wheel-shaped optomechanical microresonators on a silicon-on-insulator wafer and used optomechanical transduction for detecting their mechanical *Q* factors. Under the guidance of theoretical analysis and numerical simulation, we varied the parameter *r*_*x*_ for different resonator devices while keeping the following structural parameters fixed: *d* = 5 μm, *r*_*s*_ = 14.7 μm, *r*_*y*_ = 18.7 μm, and *R* = 26.1 μm. Figure 3a shows scanning electron microscope images of a fabricated device. Note that the wheel-shaped optomechanical microresonator also supports optical whispering-gallery modes circulating around its outer periphery, which were employed to detect the thermomechanical vibration of the resonator via optomechanical transduction. We also fabricated a bus waveguide in close proximity of the resonator for coupling light into and out of the resonator. The inset of Fig. 3a is a close-up showing the details in the coupling region of the resonator and bus waveguide. Figure 3b shows the experimental setup for device characterization. Figure 3c plots a measured optical transmission spectrum of the resonator, where the dips correspond to the optical whispering-gallery modes in different orders. Figure 3d is a close-up of a dip at ~1558.4 nm, which shows that the loaded optical *Q* factor is 2.3 × 10^5^.

**Fig. 4:**
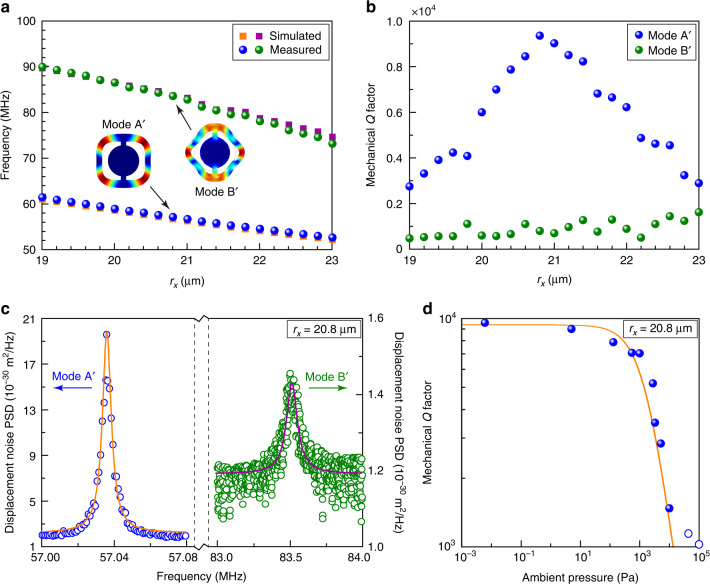
Experimental demonstration of mechanical BIC in an optomechanical microresonator. **a** Simulated and measured frequencies of modes A' and B' as a function of *r*_*x*_. **b** Measured mechanical *Q* factors of modes A' and B' as a function of *r*_*x*_ under the ambient pressure of 6.0 × 10^−3^ Pa. **c** Measured displacement noise power spectral density (PSD) of modes A' and B' from the device with *r*_*x*_ = 20.8 μm under the ambient pressure of 6.0 × 10^−3^ Pa. The blue (green) open circles represent the measured data points for mode A' (B'), and the orange (purple) line is the corresponding Lorentzian fit. **d** Measured mechanical *Q* factor of mode A' from the device with *r*_*x*_ = 20.8 μm as a function of the ambient pressure. The data collected below (above) 10^4 ^Pa are marked in blue dots (open circles). The orange curve plots a theoretical fit for the blue data points (below 10^4 ^Pa) based on Eq. (5)

To experimentally verify the mechanical BIC, we measured the wheel-shaped optomechanical microresonators in a vacuum chamber, which could provide an ambient pressure from 1.0 × 10^5^ to 6.0 × 10^−3^ Pa for the devices. Figure 4a plots the simulated and measured frequencies of modes A' and B' (modal profiles in Fig. 4a insets) for devices with different *r*_*x*_. The simulated results are extracted directly from the rightmost plot of Fig. 2c. The measured results agree well with the simulated results, which confirms the existence of the two modes. Figure 4b plots the mechanical *Q* factors of modes A' and B' as a function of *r*_*x*_ measured at the ambient pressure of 6.0 × 10^−3^ Pa. Mode A' achieves its maximal mechanical *Q* factor of 9453 at *r*_*x*_ = 20.8 μm, which agrees with the simulated results in Fig. 2d. Therefore, we confirm attainment of the mechanical BIC in our fabricated optomechanical microresonators. Figure 4c shows the measured displacement noise power spectral density of modes A' and B' at *r*_*x*_ = 20.8 μm where the BIC is achieved. The Lorentzian fitting of these spectra provides the mechanical *Q* factor of 9453 for mode A' and 882 for mode B'. The measured mechanical *Q* factor of mode A' at the BIC point is lower than the simulated value in Fig. 2d. It should be noted that the strategy of engineering a Friedrich–Wintgen BIC can only be used for eliminating the clamping loss. The other loss mechanisms such as air damping loss and material loss cannot be reduced effectively by the structural engineering and modal control^40,41^. Next, we investigated the residual loss in our BIC device (*r*_*x*_ = 20.8 μm) where the clamping loss has been completely eliminated. To this end, we measured its mechanical *Q* factor under different ambient pressures in the vacuum chamber, with the results shown in Fig. 4d. The mechanical *Q* factors for devices with other *r*_*x*_ values at different ambient pressures can be found in Supplementary Information, Section S6. Figure 4d shows that the mechanical *Q* factor decreases as the ambient pressure increases and this effect becomes more pronounced when the ambient pressure is above 1 Pa, which indicates that air damping loss was the main loss mechanism. Since material loss usually depends on temperature and maintains constant at a given temperature, e.g., room temperature in our experiment, we can express the mechanical *Q* factor as

5 $$Q^{ - 1} = Q_{{{\mathrm{0}}}}^{ - 1} + Q_{{{{\mathrm{ext}}}}}^{ - 1} = Q_{{{\mathrm{0}}}}^{ - 1} + C^{ - 1}P$$

where *Q*_0_, *Q*_ext_, and *P* are the intrinsic *Q* factor, extrinsic *Q* factor, and the ambient pressure, respectively. *C* is a proportionality constant defined as $\rho hf\sqrt {\pi ^3RT/8M}$, where *ρ*, *h*, *f*, *R*, *T*, and *M* are the material mass density, resonator thickness, mechanical frequency, ideal gas constant, temperature, and molar mass of air, respectively^42^. With *ρ* = 2329 kg m^−3^, *h* = 220 nm, *f* = 57 MHz, *R* = 8.31 J K^−1^ mol^−1^, *T* = 300 K, and *M* = 28.97 g mol^−1^, *C* has the theoretically calculated value of 1.69 × 10^7^ Pa. Note that the above expression for the mechanical *Q* factor in Eq. (5) applies only to a relatively low ambient pressure where the free-molecular-flow approximation is valid. Under a high pressure, the air-damping-dominated *Q*_ext_ follows a *P*^−1/2^ dependence^42^. Therefore, we fitted the experimental results at the ambient pressure below 10^4^ Pa based on Eq. (5), obtaining the orange curve shown in Fig. 4d with the fitted *C* being 1.49 × 10^7^ Pa, which agrees well with the theoretically calculated value.

## Discussion

In summary, we experimentally realized a BIC in an individual optomechanical microresonator, which provides a new strategy of phonon trapping in micromechanical structures with dissipation channels. By breaking the azimuthal symmetry, we introduced coupling between two dissipative mechanical modes of a wheel-shaped microresonator for making destructive interference between the dissipation channels. As a result, we obtained a Friedrich–Wintgen BIC with zero clamping loss, and achieved a mechanical *Q* factor of ~10^4^ in the very high frequency band at room temperature from a wheel-shaped optomechanical microresonator with its supporting rods’ width as large as 5 μm. To obtain high-*Q* resonances in individual micromechanical resonators, the conventional wisdom relies on minimizing the size of the supporting structures which renders the fabricated mechanical device fragile. Defying the conventional wisdom, our strategy applies to robust mechanical structures, which not only substantially reduces device fabrication difficulty but also enables device operation in versatile environments for broader application areas. Our experimental results open a new way of obtaining high-*Q* micro- and nanomechanical resonators, which will inspire plenty of applications in interdisciplinary research areas such as electromechanics, optomechanics, and quantum physics.

## Materials and methods

### Simulation

A finite-element method was adopted to simulate the mechanical modes in commercial software COMSOL. The following parameters were used in the simulation model: silicon’s Young’s modulus *E* = 150 GPa, Poisson’s ratio *ν* = 0.28, and mass density *ρ* = 2329 kg m^−3^; silicon oxide’s Young’s modulus *E* = 70 GPa, Poisson’s ratio *ν* = 0.17, and mass density *ρ* = 2200 kg m^−3^. A 2-μm-thick perfectly matched layer was placed at the bottom of the substrate for analysis of the mechanical loss. More details of mechanical simulation can be found in Supplementary Information, Section S4.

### Fabrication

The devices were fabricated with CMOS-compatible processes on a standard silicon-on-insulator wafer, where the thicknesses of the top silicon device layer and the buried silicon oxide layer are 220 nm and 3 μm, respectively. The patterns of the optomechanical microresonator, bus waveguide, and grating couplers (not shown in Fig. 3) were defined by high-resolution electron-beam lithography in an electron-beam resist (ZEP520A). Then, the patterns in the electron-beam resist were transferred to the top silicon device layer by plasma dry etching with SF_6_/C_4_F_8_ chemistry. Next, a step of photolithography was performed to define the areas to be exposed for wet etching. After that, the optomechanical microresonators were released from the substrate by wet etching in a buffered oxide etchant. Finally, the devices were dried in a critical point dryer to prevent stiction.

### Measurement

The fabricated devices were placed inside a vacuum chamber, in which the pressure could be varied from 1.01 × 10^5^ to 6.0 × 10^−3^ Pa. A laser beam from a tunable semiconductor laser (TSL) was sent through a fiber polarization controller (FPC) and a variable optical attenuator (VOA) before it was coupled into the bus waveguide of the device under test (DUT) via an input grating coupler. The laser beam was further coupled into the optomechanical microresonator to detect its thermomechanical vibration via optomechanical transduction (see details in Supplementary Information, Section S3). To obtain the intrinsic mechanical *Q* factor, the laser beam was attenuated by using the VOA such that the dynamic backaction from optomechanical interaction was negligible in the resonator (See Supplementary Information, Section S6). The light coupled out of the DUT was first amplified by a low-noise erbium-doped fiber amplifier (EDFA) and then collected by a photodetector (PD). Then, the optical signal carrying the mechanical modal information of the optomechanical microresonator was converted into the electrical domain. The converted electrical signal was received by a signal analyzer for producing the power spectral density. The mechanical *Q* factors were obtained by fitting the mechanical resonant peaks in the measured power spectral density with a Lorentzian line shape (see details in Supplementary Information, Section S3).

## Supplementary information

Supplementary Information

## Data Availability

The data that support the findings of this study are available from the corresponding author upon reasonable request.

## References

[1] Krause AG (2012). A high-resolution microchip optomechanical accelerometer. Nat. Photonics.

[2] Wallucks A (2020). A quantum memory at telecom wavelengths. Nat. Phys..

[3] Riedinger R (2018). Remote quantum entanglement between two micromechanical oscillators. Nature.

[4] Ghadimi AH (2018). Elastic strain engineering for ultralow mechanical dissipation. Science.

[5] MacCabe GS (2020). Nano-acoustic resonator with ultralong phonon lifetime. Science.

[6] Anetsberger G (2008). Ultralow-dissipation optomechanical resonators on a chip. Nat. Photonics.

[7] Cole GD (2011). Phonon-tunnelling dissipation in mechanical resonators. Nat. Commun..

[8] Von Neuman J, Wigner E (1929). On some peculiar discrete eigenvalues. Phys. Z..

[9] Marinica DC, Borisov AG, Shabanov SV (2008). Bound states in the continuum in photonics. Phys. Rev. Lett..

[10] Zou CL (2015). Guiding light through optical bound states in the continuum for ultrahigh-*Q* microresonators. Laser Photonics Rev..

[11] Yu ZJ (2019). Photonic integrated circuits with bound states in the continuum. Optica.

[12] Bulgakov EN, Sadreev AF (2008). Bound states in the continuum in photonic waveguides inspired by defects. Phys. Rev. B.

[13] Kikkawa R, Nishida M, Kadoya Y (2019). Polarization-based branch selection of bound states in the continuum in dielectric waveguide modes anti-crossed by a metal grating. N. J. Phys..

[14] Rybin MV (2017). High-*Q* supercavity modes in subwavelength dielectric resonators. Phys. Rev. Lett..

[15] Parker R (1966). Resonance effects in wake shedding from parallel plates: some experimental observations. J. Sound Vib..

[16] Sadreev AF (2021). Interference traps waves in an open system: bound states in the continuum. Rep. Prog. Phys..

[17] Deriy I (2022). Bound states in the continuum in compact acoustic resonators. Phys. Rev. Lett..

[18] Lyapina AA (2015). Bound states in the continuum in open acoustic resonators. J. Fluid Mech..

[19] Chen Y (2016). Mechanical bound state in the continuum for optomechanical microresonators. N. J. Phys..

[20] Zhao MD, Fang KJ (2019). Mechanical bound states in the continuum for macroscopic optomechanics. Opt. Express.

[21] Ha ST (2018). Directional lasing in resonant semiconductor nanoantenna arrays. Nat. Nanotechnol..

[22] Huang C (2020). Ultrafast control of vortex microlasers. Science.

[23] Kodigala A (2017). Lasing action from photonic bound states in continuum. Nature.

[24] Tittl A (2018). Imaging-based molecular barcoding with pixelated dielectric metasurfaces. Science.

[25] Wang B (2020). Generating optical vortex beams by momentum-space polarization vortices centred at bound states in the continuum. Nat. Photonics.

[26] Hsu CW (2016). Bound states in the continuum. Nat. Rev. Mater..

[27] Bulgakov EN, Sadreev AF (2017). Propagating Bloch bound states with orbital angular momentum above the light line in the array of dielectric spheres. J. Opt. Soc. Am. A.

[28] Tong H (2020). Observation of phonon trapping in the continuum with topological charges. Nat. Commun..

[29] Plotnik Y (2011). Experimental observation of optical bound states in the continuum. Phys. Rev. Lett..

[30] Hsu CW (2013). Observation of trapped light within the radiation continuum. Nature.

[31] Koshelev K (2020). Subwavelength dielectric resonators for nonlinear nanophotonics. Science.

[32] Bogdanov AA (2019). Bound states in the continuum and Fano resonances in the strong mode coupling regime. Adv. Photonics.

[33] Huang SB (2020). Extreme sound confinement from quasibound states in the continuum. Phys. Rev. Appl..

[34] Amrani M (2021). Experimental evidence of the existence of bound states in the continuum and Fano resonances in solid-liquid layered media. Phys. Rev. Appl..

[35] Cao LY (2021). Elastic bound state in the continuum with perfect mode conversion. J. Mech. Phys. Solids.

[36] Huang LJ (2021). Sound trapping in an open resonator. Nat. Commun..

[37] Friedrich H, Wintgen D (1985). Interfering resonances and bound states in the continuum. Phys. Rev. A.

[38] Han S (2021). Extended bound states in the continuum with symmetry-broken terahertz dielectric metasurfaces. Adv. Opt. Mater..

[39] Volya A, Zelevinsky V (2003). Non-Hermitian effective Hamiltonian and continuum shell model. Phys. Rev. C..

[40] Cleland, A. N. *Foundations of Nanomechanics: From Solid-State Theory to Device Applications* (Springer, 2013).

[41] Joshi S, Hung S, Vengallatore S (2014). Design strategies for controlling damping in micromechanical and nanomechanical resonators. EPJ Tech. Instrum..

[42] Gualdino A, Chu V, Conde JP (2012). Pressure effects on the dynamic properties of hydrogenated amorphous silicon disk resonators. J. Micromech. Microeng..

