# Supplementary material for: Observation of mechanical bound states in the continuum in an optomechanical microresonator
Source: Light Sci Appl. 2022 Nov 18;11:328. doi: 10.1038/s41377-022-00971-w (PMC9674641; doi:10.1038/s41377-022-00971-w)
Supplement: Supplementary file 1 — Supplementary Information [file 41377_2022_971_MOESM1_ESM.pdf]

Supplementary Information for "Observation of mechanical bound states in the continuum in an optomechanical microresonator"

Yue Yu, Xiang Xi, and Xiankai Sun*

Department of Electronic Engineering, The Chinese University of Hong Kong, Shatin, New Territories, Hong Kong SAR, China

*Corresponding author: xksun@cuhk.edu.hk

1. Temporal coupled-mode theory for Friedrich–Wintgen BIC

Temporal coupled-mode theory is widely used for describing the interaction of two different resonances in a single resonator[S1]. Let us consider a system that possesses two leaky modes and one radiation channel. The two leaky modes with complex frequencies $\Omega_1 = \omega_1 - j\gamma_1$ and $\Omega_2 = \omega_2 - j\gamma_2$ are coupled with each other and also with an incoming wave $|s_+\rangle$ and an outgoing wave $|s_-\rangle$ in the radiation channel. The dynamic equations of this system can be expressed as

$$\frac{d\mathbf{A}}{dt} = jH\mathbf{A} + K^{\mathrm{T}}\left|s_+\right\rangle \tag{S1}$$

$$\left|s_-\right\rangle = C\left|s_+\right\rangle + D\mathbf{A} \tag{S2}$$

where $\mathbf{A} = [A_1\ A_2]^{\mathrm{T}}$ represents the amplitudes of the two modes inside the resonator. H is the system's Hamiltonian defined as

$$H = \begin{bmatrix} \omega_1 - j\gamma_1 & \kappa - j\gamma_{12} \\ \kappa - j\gamma_{21} & \omega_2 - j\gamma_2 \end{bmatrix} \tag{S3}$$

where ω_1 (γ_1) and ω_2 (γ_2) are the modal frequencies (loss rates) of the two uncoupled modes, κ is the coupling rate between the two modes induced by structural perturbations, γ_{12} and γ_{21} are the coupling coefficients induced by damping. The coupling matrices are $K = D = [d_1\ d_2]$, which represents the coupling between the resonant modes and the incoming or outgoing waves. C is the scattering matrix describing the direct coupling between the incoming and outgoing waves, which is 1 in this system with only one radiation channel[S1]. With energy conversation, we obtain

$$d_1 = \sqrt{2\gamma_1}e^{j\theta_1} \tag{S4}$$

$$d_2 = \sqrt{2\gamma_2}e^{j\theta_2} \tag{S5}$$

$$d_1^* d_2 = 2\sqrt{\gamma_1\gamma_2}e^{j(\theta_2-\theta_1)} \tag{S6}$$

where θ_i is the phase angle of d_i and $\theta_2 - \theta_1 = n\pi$. With time-reversal symmetry, we obtain

$$\gamma_{12}^2 = \gamma_{21}^2 = \gamma_1\gamma_2 \tag{S7}$$

Therefore, the Hamiltonian can be simplified as

$$H = \begin{pmatrix} \omega_1 - j\gamma_1 & \kappa - j\sqrt{\gamma_1\gamma_2} \\ \kappa - j\sqrt{\gamma_1\gamma_2} & \omega_2 - j\gamma_2 \end{pmatrix} \tag{S8}$$

which is Eq. (1) in the main manuscript. Then we obtain the eigenvalues

$$j\Omega_{\pm} = j\omega_{\pm} - \gamma_{\pm} = \frac{j(\omega_1+\omega_2)-(\gamma_1+\gamma_2)\pm\sqrt{[j(\omega_1-\omega_2)-(\gamma_1-\gamma_2)]^2+4\left(j\kappa-\sqrt{\gamma_1\gamma_2}\right)^2}}{2} \tag{S9}$$

By applying the Friedrich–Wintgen condition for a BIC[S2, S3], we obtain the resonant frequencies as

$$\Omega_{+} = \frac{\omega_1+\omega_2}{2} + \frac{\kappa(\gamma_1+\gamma_2)}{2\sqrt{\gamma_1\gamma_2}} - j(\gamma_1+\gamma_2) \tag{S10}$$

$$\Omega_{-} = \frac{\omega_1+\omega_2}{2} - \frac{\kappa(\gamma_1+\gamma_2)}{2\sqrt{\gamma_1\gamma_2}} \tag{S11}$$

As a result, the Friedrich–Wintgen condition corresponds to a scenario of loss exchange: with $\kappa > 0$, the mode with a higher frequency takes all the losses (i.e., $\gamma_+ = \gamma_1 + \gamma_2$), while the mode with a lower frequency is a BIC (i.e., $\gamma_- = 0$). In this case, the mode coupling induces a BIC for the lower-frequency hybrid mode and results in an anticrossing of the eigenvalues with the Rabi frequency $\Omega_R = \frac{\kappa(\gamma_1+\gamma_2)}{\sqrt{\gamma_1\gamma_2}}$.

2. Derivation of the Friedrich–Wintgen condition for a single resonator

Breaking the azimuthal symmetry of the wheel-shaped microresonator induces coupling between mode A and mode B, which leads to two hybrid modes: mode A′ and mode B′. The eigenvalues of the hybrid modes are expressed as $j\omega_+ - \gamma_+$ and $j\omega_- - \gamma_-$, both of which satisfy Eq. (S9) as

$$j\omega_{+} - \gamma_{+} = \frac{j(\omega_1+\omega_2)-(\gamma_1+\gamma_2)+\sqrt{[j(\omega_1-\omega_2)-(\gamma_1-\gamma_2)]^2+4(j\kappa-\gamma_{12})^2}}{2} \tag{S12}$$

$$j\omega_{-} - \gamma_{-} = \frac{j(\omega_1+\omega_2)-(\gamma_1+\gamma_2)-\sqrt{[j(\omega_1-\omega_2)-(\gamma_1-\gamma_2)]^2+4(j\kappa-\gamma_{12})^2}}{2} \tag{S13}$$

The sum and difference of Eqs. (S12) and (S13) lead to

$$j(\omega_+ + \omega_-) - (\gamma_+ + \gamma_-) = j(\omega_1 + \omega_2) - (\gamma_1 + \gamma_2) \tag{S14}$$

$$j(\omega_+ - \omega_-) - (\gamma_+ - \gamma_-) = \sqrt{\left[j(\omega_1 - \omega_2) - (\gamma_1 - \gamma_2)\right]^2 + 4(j\kappa - \gamma_{12})^2} \tag{S15}$$

At the BIC point, the radiation loss of mode A′ vanishes (i.e., $\gamma_- = 0$). Since the real and imaginary parts of the left and right sides of the two equations must be equal, we obtain

$$\omega_+ + \omega_- = \omega_1 + \omega_2 \tag{S16}$$

$$\gamma_+ = \gamma_1 + \gamma_2 \tag{S17}$$

$$-(\omega_+ - \omega_-)^2 + \gamma_+^2 = -(\omega_1 - \omega_2)^2 + (\gamma_1 - \gamma_2)^2 - 4\kappa^2 + 4\gamma_{12}^2 \tag{S18}$$

$$(\omega_+ - \omega_-)\gamma_+ = (\omega_1 - \omega_2)(\gamma_1 - \gamma_2) + 4\kappa\gamma_{12} \tag{S19}$$

Substituting Eqs. (S16), (S17), and (S19) into Eq. (S18), we obtain

$$\frac{\left[(\omega_1 - \omega_2)(\gamma_1 - \gamma_2) + 4\kappa\gamma_{12}\right]^2}{(\gamma_1 + \gamma_2)^2} = (\omega_1 - \omega_2)^2 + 4\kappa^2 \tag{S20}$$

which can be expressed as

$$[\kappa(\gamma_1 - \gamma_2) - (\omega_1 - \omega_2)\gamma_{12}]^2 = 0 \tag{S21}$$

Therefore, the Friedrich–Wintgen condition is satisfied, and the BIC mode in the wheel-shaped microresonator is a Friedrich–Wintgen BIC.

3. Optical transduction of the mechanical signals in the optomechanical microresonator

At a finite temperature, a mechanical resonator in thermal equilibrium with its environment is subject to a thermomechanical noise and thus vibrates[S4]. The mechanical vibration displacement u is frequency-dependent. The displacement noise power spectral density (PSD) $S_{uu}(\omega)$ is related to the force noise PSD S_{ff} by[S5,S6]

$$S_{uu}(\omega) = \lim_{\tau \to \infty} |u(\omega)|^2 \sim \frac{\omega_m^2 S_{ff}}{(\omega_m^2 - \omega^2)^2 + (\omega_m \omega / Q_m)^2} \tag{S22}$$

where τ is the measurement time, ω_m is the mechanical resonant frequency, and Q_m is the mechanical quality factor. For frequencies near the mechanical resonant frequency, we can make the approximation $(\omega_m^2 - \omega^2) \sim 2\omega_m(\omega_m - \omega)$ such that Eq. (S22) is simplified as

$$S_{uu}(\omega) \sim \frac{S_{ff}}{4(\omega_m - \omega)^2 + \left(\omega_m / Q_m\right)^2} \tag{S23}$$

which has a Lorentzian line shape.

Next, we describe our optical transduction approach for measuring the mechanical signals. As shown in Fig. S1a, a laser beam with frequency ω_L and power P_{in} is coupled via a bus waveguide into the optomechanical microresonator. The laser frequency ω_L is slightly detuned from a cavity resonant frequency ω_c. The small thermomechanical displacement u causes a change in the optical path length, and thus shifts the cavity resonant frequency ω_c by $\Delta\omega$. As shown in Fig. S1b, the frequency shift $\Delta\omega$ leads to a different detuning of the laser from the cavity resonant frequency, and thus modulates the optical transmission T_0 by ΔT. The high optical quality factor of the microresonator enables highly efficient transduction of mechanical displacement u to optical transmission modulation ΔT, thus allowing the small thermomechanical displacement to be detected. According to the principles of cavity optomechanics[S7], in the sideband-unresolved regime, both the cavity resonant frequency variation $\Delta\omega$ and optical transmission modulation ΔT are linear to the displacement, which can be expressed as $\Delta\omega = g_{\text{om}} \cdot u$ and $\Delta T = \Delta\omega \cdot dT/d\omega$ with g_{om} being the optomechanical coupling coefficient[S8]. Therefore, the a.c. component ΔP of the output optical power P_{out} arising from the displacement u can be expressed as

$$\Delta P(\omega) = \frac{dT}{d\omega} P_{\text{in}} g_{\text{om}} u(\omega) \tag{S24}$$

By using a photodetector with responsivity g_{pd}, the above optical power is converted into an a.c. voltage $V_m = g_{\text{pd}} \cdot \Delta P$, which is then sent into a signal analyzer for obtaining the electrical PSD:

$$\text{PSD}(\omega) = \frac{V_m^2(\omega)}{Z} = \left|\frac{dT}{d\omega}\right|^2 \frac{g_{\text{pd}}^2 g_{\text{om}}^2 P_{\text{in}}^2}{Z} \left|u(\omega)\right|^2 \tag{S25}$$

where Z is the input impedance (50 Ω) of the signal analyzer.

According to Eqs. (S23) and (S25), the optically transduced mechanical signals measured on the signal analyzer can be expressed as

$$\text{PSD}(\omega) \sim \left|\frac{dT}{d\omega}\right|^2 \frac{g_{\text{pd}}^2 g_{\text{om}}^2 P_{\text{in}}^2 S_{ff}}{Z} \frac{1}{4(\omega_m - \omega)^2 + \left(\omega_m / Q_m\right)^2} \tag{S26}$$

based on which the mechanical resonant frequency ω_m and quality factor Q_m can be extracted by using Lorentzian fitting.

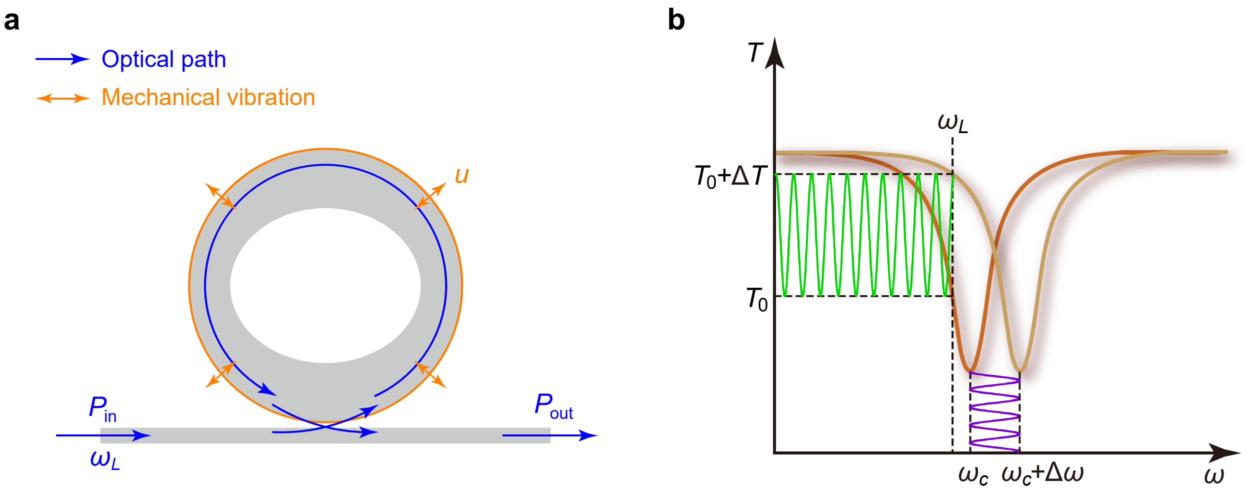

Fig. S1 a Schematic of optomechanical coupling in the wheel-shaped optomechanical microresonator**. b** Optical transduction of the mechanical signals in the optomechanical microresonator.

4. Details of mechanical simulation

To simulate the mechanical modes, we used "Solid Mechanics" module in COMSOL Multiphysics 5.5 and used "Eigenfrequency" solver to obtain the eigenfrequencies and the profiles of the corresponding eigenmodes. Below is the detailed procedure of mechanical simulation:

(1) Under tab "Home": "Add Component", then choose "3D" structure.

(2) Under tab "Geometry": Build "Geometry". We simulated two types of 3D mechanical structures, as shown in Figs. S2a and S2b. For the ring-shaped thin-plate microresonator [Fig. S2a], we either fixed h = 220 nm, R = 26.1 μm, and varied r from 18.0 to 22.0 μm or fixed h = 220 nm, R = 26.1 μm, r_y = 18.7 μm, and varied r_x from 18.0 to 22.0 μm. For the actual structure with supporting rods [Fig. S2b], the wheel-shaped microresonator was seated on a silicon oxide (SiO_2) pedestal on the silicon substrate. The shape of SiO_2 pedestal followed that of an actual device after isotropic wet etching. A 2-μm-thick perfectly matched layer (blue region) was added to encompass the finite-sized substrate for analyzing the mechanical loss. We fixed h = 220 nm, h_{SiO2} = 3 μm, h_{sub} = 10 μm, R = 26.1 μm, r_y = 18.7 μm, r_s = 14.7 μm, r_{ss} = 4.0 μm, and varied r_x from 19.0 to 23.0 μm and d from 0.5 to 8.0 μm.

(3) Under tab "Materials": "Add Material". We used two materials, silicon and silicon oxide, with the following material properties: silicon's Young's modulus E = 150 GPa, Poisson's ratio υ = 0.28, and mass density ρ = 2329 kg m^{-3}; silicon oxide's Young's modulus E = 70 GPa,

Poisson's ratio $\upsilon = 0.17$, and mass density $\rho = 2200$ kg m^{-3}.

(4) Under tab "Physics": "Add Physics", then choose "Solid Mechanics (Solid)" and apply to all domains.

(5) Under tab "Mesh": "Add Mesh", then set "Free Tetrahedral" with element size predefined as "Fine" to the entire geometry and "Build All".

(6) Under tab "Study": "Add Study", then choose "Eigenfrequency" solver. To save simulation time, we first estimated the frequency of the fundamental in-plane mode by $f_0 \sim (2\pi R)^{-1}E^{1/2}\rho^{-1/2}(1-\upsilon^2)^{-1/2} \sim 51$ MHz[S9]. Then, we set up the simulation to search for 50 eigenfrequencies around 51 MHz. We also added "Parametric Sweep" for sweeping geometric parameters. After setting up the simulation, run "Compute".

(7) Under tab "Results": We obtained the simulated eigenfrequencies and the corresponding modal displacement profiles. The mechanical Q factors were obtained from the "Expression" of "solid.Q_eig" in the interface of "Global Evaluation" under "Derived Values".

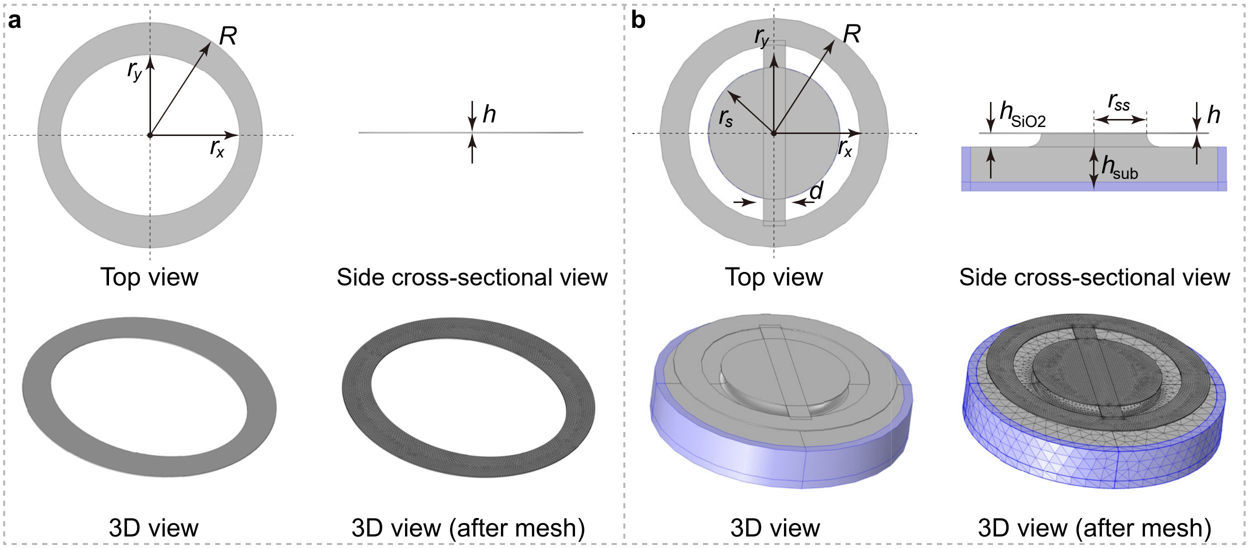

Fig. S2 **a** Different views in COMSOL of the ring-shaped thin-plate microresonator with dimension labels. **b** Different views in COMSOL of the wheel-shaped microresonator with dimension labels. The blue region denotes the 2-μm-thick perfectly matched layer. For clarity, the silicon substrate is hidden in the top view.

5. Simulated mechanical *Q* factors of the devices with different supporting rods' widths

In conventional schemes, the supporting rods for micromechanical resonators are designed as thin as possible to minimize energy dissipation to the substrate, which makes the structure fragile and

adds to fabrication difficulties. By contrast, the demonstrated mechanical BIC mode can exist for a wide range of the supporting rods' width. Figure S3 plots the simulated mechanical Q factor as a function of r_x with the supporting rods' width d varying from 1 to 8 μm. When the supporting rods' width is smaller than 3 μm, the simulated clamping-loss-limited Q factor at the BIC point is sufficiently high to be regarded as infinite. As the supporting rods' width increases, the BIC is obtained in a structure with a larger r_x and its Q factor decreases. By balancing the device performance and fabrication feasibility, we chose the rods' width to be 5 μm in the experiment.

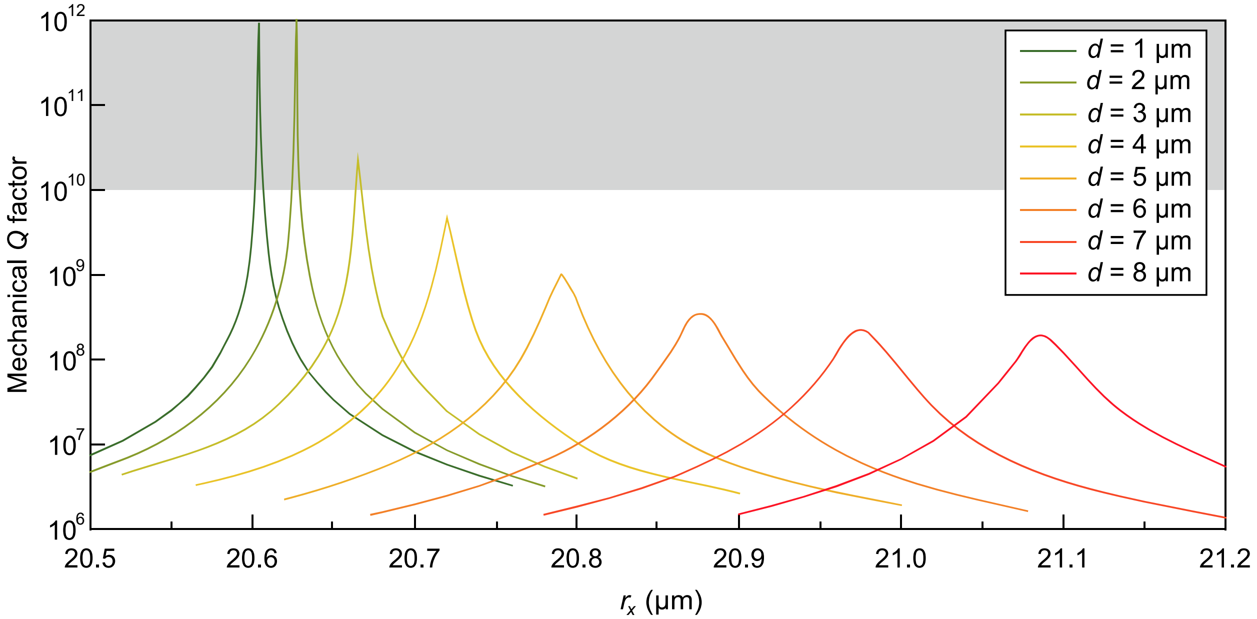

Fig. S3 Simulated mechanical Q factors of the BIC mode as a function of r_x with the supporting rods' width d varying from 1 to 8 μm. The gray area indicates the regime reaching the limit of numerical simulation, where the simulated results do not converge.

6. Additional experimental results

The mechanical energy in our devices is dissipated via air damping loss, material loss, and clamping loss. Therefore, the measured mechanical Q factor of a microresonator is a result of all the loss mechanisms. Under a high ambient pressure, air damping is the dominant loss mechanism, which determines the Q factor. Under a low ambient pressure, air damping can be neglected and the Q factor is usually limited by the clamping loss. Figure S4a shows the measured mechanical Q factors of our fabricated devices as a function of r_x under different ambient pressures. For a device with a fixed r_x, decrease of the ambient pressure leads to increase of its Q factor. Under an ambient pressure higher than 6.0×10^{-3} Pa, the Q factor near the BIC point ($r_x = 20.8$ μm) exhibits

a plateau feature in response to r_x variation, which indicates that the mechanical Q factor in this region is limited by air damping loss.

Figure S4b plots the measured thermomechanical noise spectra for the device with $r_x = 20.8$ μm under the ambient pressure of 6.0×10^{-3} Pa at different input optical power levels. The Lorentzian fitted Q factors do not vary with the input optical power, which precludes the backaction-induced heating and cooling effects during the optomechanical measurement.

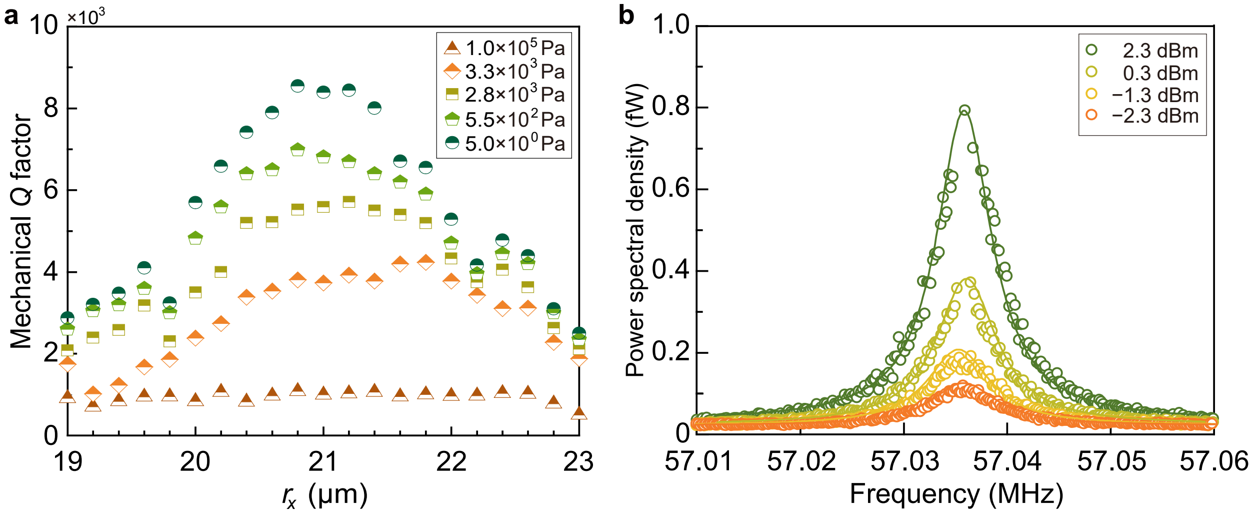

Fig. S4 **a** Measured mechanical Q factors under different ambient pressures. **b** Measured thermomechanical noise power spectra for the device with $r_x = 20.8$ μm under the ambient pressure of 6.0×10^{-3} Pa at different input optical power levels. The open circles represent the measured data, and the solid lines plot the corresponding Lorentzian fits.

References

S1. Suh, W., Wang, Z. & Fan, S. Temporal coupled-mode theory and the presence of non-orthogonal modes in lossless multimode cavities. *IEEE J. Quantum Electron.* **40**, 1511–1518 (2004).

S2. Kikkawa, R., Nishida, M. & Kadoya, Y. Polarization-based branch selection of bound states in the continuum in dielectric waveguide modes anti-crossed by a metal grating. *New J. Phys.* **21**, 113020 (2019).

S3. Volya, A. & Zelevinsky, V. Non-Hermitian effective Hamiltonian and continuum shell model. *Phys. Rev. C* **67**, 054322 (2003).

S4. Poot, M. & van der Zant, H. S. Mechanical systems in the quantum regime. *Phys. Rep.* **511**, 273–335 (2012).

S5. Aspelmeyer, M., Kippenberg, T. J., & Marquardt, F. Cavity optomechanics. *Rev. Mod. Phys.* **86**, 1391 (2014).

S6. Shanmugan, K. S. & Breipohl, A. M. *Random Signals: Detection, Estimation and Data Analysis*. John Wiley & Sons (1988).

S7. Kippenberg, T. J. & Vahala, K. J. Cavity opto-mechanics. *Opt. Express* **15**, 17172–17205 (2007).

S8. Krause, A. G., Winger, M., Blasius, T. D., Lin, Q. & Painter, O. A high-resolution microchip optomechanical accelerometer. *Nat. Photonics* **6**, 768–772 (2012).

S9. Stephenson, C. V. Radial vibrations in short, hollow cylinders of barium titanate. *J. Acoust. Soc. Am.* **28**, 51–56 (1956).
